# Editorial: Clinical Application of Stereotactic Body Radiotherapy (SBRT): Cranium to Prostate

**DOI:** 10.3389/fonc.2015.00266

**Published:** 2015-12-07

**Authors:** John A. Vargo, Dwight E. Heron

**Affiliations:** ^1^Radiation Oncology, University of Pittsburgh Cancer Institute, Pittsburgh, PA, USA

**Keywords:** SBRT, SRS, reirradiation, prostate cancer, head and neck cancer, lung cancer, adrenal metastases, brain metastases

Stereotactic radiosurgery is a relatively recent radiation technique initially developed using a frame-based system in 1949 by a Swedish neurosurgeon, Lars Leksell, for lesions not amendable to surgical resection. Radiosurgery is founded on principles of extreme radiation dose escalation, afforded by precise dose delivery with millimeter to submillimeter accuracy. Building upon the success of frame-based radiosurgery techniques, which were limited to cranial tumors and invasive head-frame placement, advances in radiation delivery and image guidance have led to the development of stereotactic body radiotherapy (SBRT). SBRT allows for frameless delivery of dose distributions akin to frame-based cranial stereotactic radiosurgery to both cranial and extracranial sites and has emerged as an important treatment strategy for a variety of cancers from the cranium to prostate.

In this research topic, we present a compendium of scientific papers that highlight the forefront of clinical applications of SBRT. This collection of papers showcase the wide application of SBRT for primary cancers often in patient populations in whom conventional treatment strategies are either not possible anatomically, fraught with risk due to medical comorbidities, or present significant threats to patient quality of life. This includes the primary treatment for elderly patients with inoperable head and neck cancers, medically inoperable early-stage non-small cell lung cancer, adrenal metastases, and early-stage organ confined prostate cancer. Through stereotaxy, SBRT limits the volume of tissue that is irradiated which is especially important when considering reirradiation for recurrent tumors; this is highlighted through the collection with papers discussing SBRT for reirradiation of primary brain tumors, skull-base, and parenchymal brain metastases, and gynecologic tumors. Finally, as a number of papers herein highlight, SBRT both due to its short overall treatment time, minimal acute side effects, and unique underlying radiobiological effects, holds the potential for integration with novel systemic therapies aimed at improving outcomes and even potentially engaging the immune system in the oncologic armamentarium. This collection could, thus, serve as an invaluable resource for the growing breadth of SBRT application as physicians continue the relentless pursuit of tackling some of the most challenging cases in oncology.

## Author Contributions

Both JV and DH were responsible for drafting and finalizing manuscript.

## Conflict of Interest Statement

The authors declare that the research was conducted in the absence of any commercial or financial relationships that could be construed as a potential conflict of interest.

